# The Hunger Vital Sign Identifies Household Food Insecurity among Children in Emergency Departments and Primary Care

**DOI:** 10.3390/children6100107

**Published:** 2019-10-02

**Authors:** Rajender K. Gattu, Grace Paik, Yan Wang, Prema Ray, Richard Lichenstein, Maureen M. Black

**Affiliations:** 1 Division of Pediatric Emergency Medicine, Department of Pediatrics, University of Maryland School of Medicine, Baltimore, MD 21201, USAprema.ray1@gmail.com (P.R.); rlichenstein@som.umaryland.edu (R.L.); 2 Division of Growth & Nutrition, Department of Pediatrics, University of Maryland School of Medicine, Baltimore, MD 21201, USA; gpaik06@gmail.com (G.P.); yan.wang@som.umaryland.edu (Y.W.); 3 RTI International, Research Triangle Park, NC 27709, USA

**Keywords:** food insecurity, adverse child health, emergency department, primary care, young children, hunger vital sign

## Abstract

This study aimed: (1) to examine the sensitivity and specificity of the 2-item Hunger Vital Sign against the 18-item Household Food Security Survey Module (HFSSM) in identifying young children in food insecure households in emergency department and primary care sites and (2) to examine associations between food insecurity and adverse health conditions. We conducted cross-sectional surveys from 2009–2017 among 5039 caregivers of children age <48 months. We measured adverse child health by caregiver-reported perceived health, prior hospitalizations, and developmental risk (Parents’ Evaluation of Developmental Status). Analyses were conducted using covariate-adjusted logistic regression. Sensitivity and specificity of the Hunger Vital Sign against the HFSSM were 96.7% and 86.2%. Using the HFSSM, children in the emergency department had a 28% increase in the odds of experiencing food insecurity, compared to children in primary care, aOR = 1.28, 95% Confidence Interval (CI) = 1.08–1.52, *p* = 0.005. Using the Hunger Vital Sign, the increase was 26%, aOR = 1.26, 95% CI = 1.08–1.46, and *p* = 0.003. The odds of children’s adverse health conditions were significantly greater in food insecure households, compared to food secure households, using either HFSSM or the Hunger Vital Sign. Screening for food insecurity with the Hunger Vital Sign identifies children at risk for adverse health conditions in both primary care and emergency department sites, and can be used to connect families with resources to alleviate food insecurity.

## 1. Introduction

Household food insecurity, lacking access to enough food for an active, healthy life for all household members [1], is a national public health problem with negative health consequences throughout life [2]. In 2017, households with children under age six years had higher rates of food insecurity, compared to households without children (16.4% vs. 10.1%) [1]. Access to nutritious food is particularly critical early in life during the period of rapid growth and brain development. Families with food insecurity often substitute low nutrient dense food for nutritious food to assuage hunger [3]. Young children raised in food insecure households are at risk for adverse health consequences, including perceived fair/poor health [4,5]; prior hospitalizations [4]; developmental risk [5,6]; cognitive impairment [7]; and behavioral dysfunction and emotional distress [8]. Inadequate nutritional intake can increase children’s vulnerability to future adverse chronic conditions, such as obesity, diabetes and cardiovascular disease [9]. When the parental stress and depression that are often associated with household food insecurity are also considered, the vulnerability associated with food insecurity can last throughout life [9,10], leading to higher healthcare costs [11].

Household food insecurity is often invisible, as young children do not necessarily have compromised growth [12,13]. Unless providers specifically ask about food insecurity, they may not recognize children in food insecure households. A 2-item screener, known as the Hunger Vital Sign, has been developed and validated against the gold standard Household Food Security Survey Module (HFSSM) showing high sensitivity and specificity among young children [14]. The validity of the 2-item screener has been extended to adolescents and adults [15,16]. In 2015, the American Academy of Pediatrics issued a policy statement recommending that pediatricians screen families with children for food insecurity, and implement referrals as needed [17]. The policy statement does not mention screening in emergency departments (ED). 

Recent reports indicate increases in ED use among children, primarily Medicaid beneficiaries [18], suggesting that EDs serve as a safety net for low-income families. If rates of household food insecurity are high among children in EDs, there may be missed opportunities to connect families with resources to reduce food insecurity. In addition, if food insecurity is associated with adverse health conditions, there may also be missed opportunities to identify and improve children’s adverse health conditions. 

The purpose of this study is to examine the sensitivity and specificity of the 2-item Hunger Vital Sign against the gold standard 18-item HFSSM in identifying household food insecurity among young children in an ED and primary care, and to compare the associations between food insecurity and adverse health conditions using both the Hunger Vital Sign and the HFSSM. A finding that the Hunger Vital Sign is effective in identifying food insecure households of young children in the ED and that food insecurity among households of young children is associated with adverse health conditions, measured with either the HFSSM or the Hunger Vital Sign and across sites would provide additional evidence that the Hunger Vital Sign can (and should) be used to screen for food insecurity across multiple sites, including EDs. 

## 2. Materials and Methods

### 2.1. Sample

Data were obtained from Children’s HealthWatch, an ongoing multi-center study that monitors how social and economic factors relate to children’s health and wellbeing in urban medical centers [19]. Data were collected between April 2009 and December 2017 through a series of cross-sectional surveys administered in a pediatric ED and a primary care site in a large urban medical center serving the same low-income urban community. Participants were caregivers of children younger than 48 months who were seeking medical care for their children. Eligibility included low-income (lack of private insurance was used as an indicator), comprehension of English (fewer than 6% of patients do not comprehend English), state residency, and knowledge of the child’s health and household. Caregivers of critically ill or injured children were excluded. Institutional review board approval was obtained prior to data collection and renewed annually. After obtaining informed consent, trained research assistants interviewed caregivers in private settings to elicit caregivers’ verbal responses to a computer-based survey. 

### 2.2. Measures

The Children’s HealthWatch survey [19] includes the following measures:

#### 2.2.1. Demographics

Caregivers provided information on their age, self-identified race/ethnicity, country of origin, marital and employment status, highest level of education, number of household members, and children’s age, sex, health insurance and breastfeeding history. 

#### 2.2.2. Food Insecurity

The 18-item HFSSM asks about food insecurity during the last 12 months. According to established procedures from the United States Department of Agriculture Economic Research Service, households are classified as food insecure if they indicate that they lacked access to enough food for an active, healthy life for all household members in the past 12 months. A household was classified as food insecure if three or more questions were endorsed as sometimes or often true vs. never true [1].

Food insecurity was also measured by the Hunger Vital Sign [19], the first two items from the HFSSM: “Within the past 12 months we worried whether our food would run out before we got money to buy more” and “Within the past 12 months the food we bought just didn’t last and we didn’t have money to get more.” Households of caregivers who endorsed either “Always true” or “Sometimes true” to either or both questions were classified as food insecure. 

The dependent measures were caregivers’ perception of child health, and report of hospitalizations and developmental risk.

#### 2.2.3. Perceived Child Health

Children’s perceived health was measured with a validated question [20,21] from the National Health and Nutrition Examination Survey: “In general, would you say your child’s physical health is …? Excellent, good, fair, or poor”. Responses were categorized as excellent/good or fair/poor. 

#### 2.2.4. Child Hospitalizations

Caregivers reported the number of inpatient hospitalizations since birth. 

#### 2.2.5. Developmental Risk

Caregivers reported on their child’s development using the Parents’ Evaluation of Developmental Status (PEDS), a validated 10-item caregiver-reported screening instrument [22,23]. Caregivers reported any concerns (no, yes, or a little) in response to questions about the child’s development in expressive and receptive language, fine and gross motor, behavior, social/emotional, self-help, and school performance, and to open-ended questions about concerns in global/cognitive development and “other”. Children with two or more concerns were classified at developmental risk [22,23].

### 2.3. Statistical Analysis 

#### 2.3.1. Food Insecurity in the ED and Primary Care

Chi-square tests were conducted to compare the prevalence of demographic characteristics among caregivers of young children seeking services, comparing households classified as food insecure versus food secure and comparing caregivers in the ED versus primary care. 

#### 2.3.2. Sensitivity and Specificity of the Hunger Vital Sign 

Sensitivity was calculated as the number of households classified as food-insecure by the Hunger Vital Sign divided by the number of households classified as food-insecure by the HFSSM. Specificity was calculated as the number of households classified as food secure by the Hunger Vital Sign divided by the number of number of households classified as food secure by the HFSSM. Positive predictive value was calculated as the number of children identified as food secure by both the HFSSM and the Hunger Vital Sign divided by the total number of children identified as food secure by the Hunger Vital Sign and negative predictive value was calculated by the number of children identified as food insecure by both measures divided by the total number identified as food insecure by the Hunger Vital Sign. 

#### 2.3.3. Adverse Child Health Conditions by Food Security and Site

We examined the prevalence of adverse health conditions using chi-square tests and then conducted logistic regressions to examine whether the odds of adverse health conditions varied by household food security status and by site. We began with crude analyses without covariates and then adjusted by covariates, including maternal age, education, race, marital status, and employment, and child age and birthweight. Interactions between site and food insecurity in the HFSSM and the ED were included to examine site differences in the relations. 

SPSS 22.0 statistical software (Manufacturer, City, US State abbrev. if applicable, Country) was used. Given the number of tests conducted, we set statistical significance at *p* < 0.01.

## 3. Results

Of the 6519 children assessed in 2009–2017, 1480 were excluded (861 had private insurance and 619 had acute health problems, including 187 who were admitted to the hospital), leaving an analytic sample of 5039.

As shown in Table 1, 90% of caregivers were mothers (6% fathers, 3% grandparents, and 1% other); 84% of caregivers were over 21 years-of-age; 89% were African American; 78% completed high school, including 32% with technical or post-high school education; 43% were employed, and 77% were single, separated, divorced, or widowed. Over half of the children were males (53%), 74% were younger than 24 months, and 17% had a history of low birthweight. Eight percent of caregivers perceived their child’s health as fair/poor, 20% of children had a hospitalization history, and 16% were at developmental risk. 

Table 1 also shows a higher prevalence of maternal employment and a higher prevalence of children age <24 months among caregivers in food secure, compared to food insecure households. In addition, based on the Hunger Vital Sign, but not HFSSM, the prevalence of caregivers who had completed high school and who were not married was higher in food secure compared to food insecure households. 

In the ED, compared to primary care, Table 1 shows a higher prevalence of caregivers younger than age 21, of African American race, of not being married, and of having a child aged 25–47 months. There were no differences between the ED and primary care in the prevalence of children’s birth weight or in maternal education or employment.

### 3.1. Food Insecurity in the ED and Primary Care

As shown in Table 1, based on the HFSSM, the prevalence of household food insecurity was higher in the ED (22.7%), compared to primary care (17.9%) (*p* < 0.001). Similarly, based on the Hunger Vital Sign, the prevalence of household food insecurity risk was higher in the ED (32.9%), compared to 27.7% in primary care, *p* < 0.001.

As shown in Table 2, based on the HFSSM, the crude odds ratio for food insecurity in the ED was significantly greater than in primary care, indicating a 35% increase in the odds of young children in the ED experiencing food insecurity, compared to children in primary care. After adjusting for covariates, the odds ratio for food insecurity in the ED was significant, but attenuated and indicated a 28% increase in the odds of young children in the ED experiencing food insecurity, compared to children in primary care.

Table 2 also shows that the crude odds ratio for food insecurity in the ED based on the Hunger Vital Sign was significantly greater than in primary care, indicating a 28% increase in the odds of young children in the ED experiencing food insecurity, compared to children in primary care. After adjusting for covariates, the odds ratio for food insecurity in the ED based on the Hunger Vital Sign was similar, indicating a 26% increase in odds of young children in the ED experiencing food insecurity, compared to children in primary care.

### 3.2. Sensitivity and Specificity of the Hunger Vital Sign

The sensitivity of the Hunger Vital Sign against the gold standard, HFSSM, was 96.7% and the specificity was 86.2% (see Table 3). Among the children identified as household food insecure by the HFSSM, 96.7% were also identified as food insecure by the Hunger Vital Sign and 3.3% were misclassified as food secure. Of the children were identified as food secure by the HFSSM, 86.2% were also identified as food secure by the Hunger Vital Sign and 13.8% were misclassified as food insecure. Positive predictive value, the probability that children classified as food secure on the Hunger Vital Sign are food secure based on the HFSSM is 65.7% and negative predictive value, the probability that children classified as food insecure on the Hunger Vital Sign are food insecure on the HFSSM, is 99.0%. 

### 3.3. Adverse Child Health Conditions by Food Security and Site 

As shown in Table 1, the prevalence of children with adverse health conditions was higher in the ED, compared with primary care, including children perceived to be experiencing fair/poor health (8.8% vs. 5.5%, *p* < 0.001) and prior hospitalizations (22.6% vs. 13.9%, *p* < 0.001). There were no site differences in the prevalence of children with developmental risks (16.1% vs. 17.9%, *p* = 0.237). After covariate adjustment, Table 4 shows that children in the ED had 63% higher odds of being perceived in fair/poor health (aOR = 1.63, 95% CI: 1.22–2.17, *p* < 0.001) and 65% higher odds of prior hospitalization, compared to children in primary care (aOR = 1.65, 95% CI: 1.36–1.99, *p* < 0.001). Site was not related to developmental risk in either unadjusted or adjusted analyses. 

Children in food insecure households, defined by HFSSM, had 72% greater odds of fair/poor perceived health (aOR = 1.72, 95% CI: 1.37–2.18, *p* < 0.001), 37% greater odds of prior hospitalization (aOR = 1.37, 95% CI: 1.16–1.62, *p* < 0.001), and 46% greater odds of developmental risk (aOR = 1.46, 95% CI: 1.19–1.78, *p* < 0.001), compared to children in food secure households after covariate adjustment, as shown in Table 4. Interactions between site and food insecurity defined by HFSSM were not significant for developmental risk and fair/poor perceived health, but the interaction was significant for prior hospitalization (*p* = 0.008). Stratified analyses showed that the relation between food insecurity and prior hospitalization was significant in primary care (aOR = 2.09, 95% CI: 1.42–3.08, *p* < 0.001), but not in the ED (aOR = 1.22, 95% CI: 1.02–1.47, *p* = 0.033, not shown in the table). 

Analyses were repeated defining food insecurity by the Hunger Vital Sign, as shown in Table 4. Findings were similar. Children in food insecure households, defined by the Hunger Vital Sign, had 53% greater odds of fair/poor perceived health (aOR = 1.53, 95% CI: 1.23–1.91, *p* < 0.001), 28% greater odds of prior hospitalization (aOR = 1.28, 95% CI: 1.10–1.48, *p* = 0.002), and 44% greater odds of developmental risk (aOR = 1.44, 95% CI: 1.20–1.73, *p* < 0.001), compared to children in food secure households after covariate adjustment. There was a significant interaction for prior hospitalization (*p* = 0.005), but not for fair/poor health or developmental risk. Stratified analyses showed that the relation between food insecurity and prior hospitalization was significant in primary care (aOR = 1.99, 95% CI: 1.40–2.84, *p* < 0.001), but not in the ED (aOR = 1.16, 95% CI: 0.98–1.37, *p* = 0.083, not shown in the table).

## 4. Discussion

This research yields three findings related to household food insecurity among young children under age four years in health care sites. First, based on the HFSSM, 22% of the children in the ED were living in a food insecure household, a rate substantially higher than in primary care, and higher than the national rate for households with children under age six years [1], illustrating the high likelihood of young children in EDs experiencing household food insecurity.

Second, children in food insecure households were at increased risk for perceived fair/poor health, prior hospitalizations, and developmental risk, across both health care sites, illustrating the vulnerabilities associated with food insecurity. These findings contribute to the evidence linking food insecurity and adverse health conditions among young children reported previously [4,5,6,24]. Inexpensive foods that are low in nutrients and high in energy may increase children’s vulnerability to nutritional deficiencies and associated adverse health conditions [25]. In addition, the parenting stress and anxiety associated with food insecurity has been associated with non-responsive feeding behavior [26]. Non-responsive parenting skills may contribute to children’s developmental problems. 

An elevated risk of food insecurity has been reported among children with special health care needs [27]. Although it is not clear why the odds of prior hospitalization among children in food secure households were significant in the primary care site, but not in the ED, a possible explanation is that the primary care site provides specialty care for children with special health care needs. Thus, the association between food insecurity and prior hospitalization in primary care may represent increased vulnerability among children with special health care needs. 

Third, the Hunger Vital Sign [19], was effective in identifying children in food insecure households and in replicating the analyses linking food insecurity with adverse child health conditions conducted with the HFSSM. The sensitivity and specificity of the Hunger Vital Sign are consistent with prior findings [14,15,16]. The higher rates of food insecurity in the ED and primary care based on the Hunger Vital Sign, compared to the HFSSM, occur because children classified as living in marginal food secure households on the HFSSM (endorsement of one or two questions) are classified as food insecure using the Hunger Vital Sign. Children in marginal food secure households have been shown to be at risk for adverse child health conditions [28]. Thus, identifying children through the Hunger Vital Sign could lead to closer attention to previously unrecognized adverse health conditions. These findings suggest that screening for household food insecurity in both EDs and primary care will uncover additional children living in food insecure households.

The Hunger Vital Sign is easy to administer and is increasingly being incorporated into electronic medical records (EMR) [29]. Efforts to increase screening for household food insecurity have been effective in primary care [30] and in other clinics serving low-income families [31]. Providers are developing innovative strategies to connect food insecure families with services [32]. In addition to federal nutritional services, such as the Supplemental Nutritional Assistance Program (SNAP) and the special supplemental nutrition program for Women, Infants and Children (WIC), families can benefit from local resources, including food pantries and food banks [30]. Hennepin County Medical Center in Minnesota created an innovative referral system integrated into the EMR [33]. With family’s consent, contact information on food insecure families is auto-faxed to a partner food bank. Trained outreach food bank staff make telephone contact with families and provide application assistance for food assistance programs (e.g., SNAP) and geographically individualized information about WIC, neighborhood food shelves, produce distributions, summer feeding sites and community meals. In 2015, 64% of the 1003 patient EMR-based referrals were successfully contacted and 82% were connected with at least one new form of food assistance. Of persons contacted and not currently enrolled in SNAP, applications were completed for 67%, 26% were found to be ineligible, and 7% declined to apply [33].

Emergency Departments have a demonstrated record of effective health screening, including brief survey tools for mental health conditions such as suicide [34]. Adolescents in EDs have been shown to accept screenings for HIV [35], enabling clinicians to implement recommendations from the Centers for Disease Control and Protection. Screening procedures have enabled clinicians to provide linkages to hospital, outpatient, and community resources for patients who may have otherwise had limited access to needed services. Integrating the Hunger Vital Sign into the EMR during triage may be a cost-efficient method to flag patients at risk of food insecurity and in need of resources. 

There are several methodological limitations to consider. First, the sample included health-seeking caregivers and their young children from one urban medical center. However, the sample size was large, covered multiple years, and addressed very young children, a population known to be vulnerable to threats of food insecurity. Second, information about food security and children’s health conditions was gathered from caregiver-report. However, validated measures were used and data were collected by trained research assistants, reducing, but not eliminating concerns about recall and shared method bias. Third, all caregivers received public health insurance and were from the same urban community, limiting the generalizability of the findings. Demographic characteristics, including maternal age, race, marital status and employment, and child age and sex differed between the ED and primary care. However, analyses were adjusted for these demographic differences. Finally, the sample was limited to children under four years of age. Although young children are vulnerable to household food insecurity [4,5], they have access to nutritional assistance programs such as WIC and therefore may be less vulnerable than older children. 

## 5. Conclusions

EDs provide services to highly vulnerable children. In comparison to children from primary care, families of children seeking care in an ED have higher rates of household food insecurity, fair/poor health, and prior hospitalizations. Across both health care sites, household food insecurity increases the odds of caregiver-perceived fair/poor health, prior hospitalizations, and developmental risk. Implementing the Hunger Vital Sign into triage in EDs identifies children at risk for adverse health conditions. Innovative strategies have shown effective ways of providing food insecure households with resources to reduce food insecurity and associated adverse health conditions. Additional research is necessary to identify ways to connect families in food insecure households to resources and to remove barriers that may prevent families from accessing food assistance programs or food resources.

## Figures and Tables

**Table 1:** Demographic information by food insecurity and health care site (*n* = 5039) ^1^.

		HFSSM			Hunger Vital Sign			Primary Care	Emergency Dept	
	*n* (%)	Food Secure *n* = 3954 (%)	Food Insecure *n* = 1082 (%)	*p*	Food Secure *n* = 3443 (%)	Food Insecure *n* = 1593 (%)	*p*	*n* = 1239 (%)	*n* = 3800 (%)	*p*
**Maternal Age**				0.026			0.069			<0.001
<21 years	816 (16.4)	662 (16.9)	152 (14.1)		578 (17.0)	236 (14.9)		266 (21.7)	550 (14.6)	
≥21 years	4174 (83.6)	3247 (83.1)	926 (85.9)		2828 (83.0)	1345 (85.1)		961 (78.3)	3213 (85.4)	
**Maternal Race**				<0.001			0.036			0.006
African American	4438 (88.8)	3517 (89.6)	918 (85.9)		3051 (89.4)	1384 (87.7)		1122 (91.3)	3316 (88.0)	
White	318 (6.4)	237 (6.0)	81 (7.6)		217 (6.4)	101 (6.4)		63 (5.1)	255 (6.8)	
Hispanic or other	240 (4.8)	170 (4.3)	70 (6.5)		146 (4.3)	94 (6.0)		44 (3.6)	196 (5.2)	
**Maternal Education**				0.014			0.002			0.488
>High school (HS)	3898 (77.5)	3089 (78.3)	808 (74.7)		2706 (78.8)	1191 (74.8)		949 (76.8)	2949 (77.7)	
Some HS or less	1132 (22.5)	857 (21.7)	273 (25.3)		729 (21.2)	401 (25.2)		287 (23.2)	845 (22.3)	
**Maternal Employment**				<0.001			<0.001			0.118
Yes	2163 (43.0)	1755 (44.5)	408 (37.7)		1558 (45.3)	605(38.0)		507 (41.1)	1656 (43.6)	
No	2867 (57.0)	2192 (55.5)	673 (62.3)		1878 (54.7)	987(62.0)		727 (58.9)	2140 (56.4)	
**Maternal Marital Status**				0.021			0.003			<0.001
Married	1170 (23.3)	889 (22.5)	280 (25.9)		758 (22.1)	411 (25.8)		357 (28.9)	813 (21.4)	
Single/Divorced/Widow	3861 (76.7)	3057 (77.5)	802 (74.1)		2679 (77.9)	1180 (74.0)		879 (71.1)	2982 (78.6)	
**Child Gender**				0.449			0.759			0.002
Male	2683 (53.2)	2116 (53.5)	565 (52.2)		1838 (53.4)	843 (52.9)		612 (49.4)	20171 (54.5)	
Female	2356 (46.8)	1838 (46.5)	517 (47.8)		1605 (46.6)	750 (47.1)		627 (50.6)	1729 (45.5)	
**Child Age**				<0.001			<0.001			<0.001
<24 months	3750 (74.4)	2989 (75.6)	758 (70.1)		2613 (75.9)	1134 (71.2)		1110 (89.6)	2640 (69.5)	
24–47 months	1289 (25.6)	965 (24.4)	324 (29.9)		830 (24.1)	459 (28.8)		129 (10.4)	1160 (30.5)	
**Child Birth Weight**				0.067			0.256			0.137
Normal (≥2500 g)	4108 (83)	3202 (82.5)	904 (84.9)		2792 (82.6)	1314 (83.9)		1004 (81.6)	3104 (83.5)	
Low (<2500 g)	841 (17)	679 (17.5)	161 (15.1)		588 (17.4)	252 (16.1)		226 (18.4)	615 (16.5)	
**HFSSM**										<0.001
Food secure	3954 (78.5)	----	----	----	----	----	----	1017 (82.1)	2937 (77.3)	
Food insecure	1082 (21.5)	----	----	----	----	----	----	221 (17.9)	861 (22.7)	
**Hunger Vital Sign**										
Food secure	3443 (68.4)	----	----	----	----	----	----	895 (72.3)	2548 (67.1)	
Food insecure	1593 (31.6)	----	----	----	----	----	----	343 (27.7)	1250 (32.9)	<0.001
**Housing Insecure**				<0.001						
Housing secure	3623 (71.9)	3127 (79.1)	493 (45.6)		----	----	----	919 (74.2)	2704 (71.2)	
Housing insecure	1416 (28.1)	827 (20.9)	589 (54.4)		----	----	----	320 (25.8)	1096 (28.8)	0.040

^1^ 3 children lacked food security data. HFSSM = Household Food Security Survey Module.

**Table 2:** Logistic regression of food insecurity based on HFSSM and Hunger Vital Sign related to health care site.

	Crude Odds Ratio 95% CI	*p*	Adjusted Odds Ratio 95% (CI)	*p*
**HFSSM**				
**Health Care Site**		<0.001		0.005
Primary Care	1 (Reference)		1 (Reference)	
Emergency Dept	1.35 (1.15–1.59)		1.28 (1.08–1.52)	
**Hunger Vital Sign**				
**Health Care Site**		0.001		0.003
Primary Care	1 (Reference)		1 (Reference)	
Emergency Dept	1.28 (1.11–1.48)		1.26 (1.08–1.46)	

Note: HFSSM = Household Food Security Survey Module. Adjusted Odds Ratios were estimated based on logistic regression models, adjusting for maternal age, race, education, marital status, and employment, and child age and birthweight.

**Table 3:** Classification of sensitivity and specificity of the Hunger Vital Sign against the HFSSM.

		HFSSM		
		Negative (Food Insecure)	Positive (Food Secure)	
**Hunger Vital Sign**	**Negative (Food Insecure)**	3408	35	NPV = 99.0%
	**Positive (Food Secure)**	546	1047	PPV = 65.7%
		Sensitivity = 96.8%	Specificity = 86.2%	

Note: PPV = Positive predictive value, NPV = Negative predictive value, HFSSM = Household Food Security Survey Module.

**Table 4:** Logistic regression of adverse health conditions related to health care site and food insecurity based on HFSSM and Hunger Vital Sign (*n* = 5039).

Perceived Child Health								
	Excellent/Good (*n* = 4629)	Fair/Poor (*n* = 402)	Crude Odds Ratio	*p*	Adjusted Model HFSSM	*p*	Adjusted Hunger Vital Sign Model	*p*
			(95% CI)		(95% CI)		(95% CI)	
**Health Care Site**				<0.001		0.001		0.001
Primary care	1167 (94.5)	68 (5.5)	1 (Reference)		1 (Reference)		1 (Reference)	
Emergency dept	3460 (91.2)	334 (8.8)	1.66 (1.27–2.17)		1.60 (1.20–2.14)		1.60 (1.20–2.13)	
**HFSSM**				<0.001		<0.001		
Food secure	3671 (93.1)	236 (6.9)	1 (Reference)		1 (Reference)		---	---
Food insecure	953 (88.5)	166 (10.5)	1.74 (1.39–2.17)		1.72 (1.37–2.18)		---	---
**Hunger Vital Sign**				<0.001				<0.001
Food secure	3203 (93.0)	277 (7.0)	1 (Reference)		---	---	1 (Reference)	
Food insecure	1421 (89.5)	125 (11.5)	1.59(1.29–1.95)		---	---	1.53 (1.23–1.91)	
**Interaction (HFSSM × site)**		---	---	---	0.77 (0.42–1.42)	0.404	---	---
**Interaction (Hunger Vital Sign × site)**		---	---	---	---	---	1.02 (0.57–1.84)	0.938
**Prior Hospitalization**								
	**No (*n* = 3994)**	**Yes (*n* = 1025)**	**Crude Odds Ratio**	***p***	**Adjusted Model HFSSM**	***p***	**Adjusted Hunger Vital Sign Model**	***p***
			**(95% CI)**		**(95% CI)**		**(95% CI)**	
**Health Care Site**				<0.001		<0.001		<0.001
Primary care	1062 (86.1)	171 (13.9)	1 (Reference)		1 (Reference)		1 (Reference)	
Emergency dept	2932 (77.4)	854 (22.6)	1.81 (1.51–2.16)		1.63 (1.35–1.97)		1.63 (1.35–1.96)	
**Food Insecurity (HFSSM)**				<0.001		<0.001		
Food secure	3185 (80.9)	752 (19.1)	1 (Reference)		1 (Reference)		---	---
Food insecure	806 (74.7)	273 (25.3)	1.44 (1.22–1.68)		1.35 (1.14–1.59)		---	---
**Hunger Vital Sign**				<0.001				0.002
Food secure	2785 (81.2)	643 (18.8)	1 (Reference)		---	---	1 (Reference)	
Food insecure	1206 (75.9)	382 (24.1)	1.37 (1.19–1.58)		---	---	1.28 (1.10–1.48)	
**Interaction (HFSSM × site)**		---	---	---	0.57 (0.37–0.86)	0.008	---	---
**Interaction (Hunger Vital Sign × site)**		---	---	---	---	---	0.58 (0.39–0.85)	0.005
**Developmental Risk**								
	**No (*n* = 3317)**	**Yes (*n* = 653)**	**Crude OR**	***p***	**Adjusted Model HFSSM**	***p***	**Adjusted Hunger Vital Sign Model**	***p***
			**(95% CI)**		**(95% CI)**		**(95% CI)**	
**Health Care Site**				0.237		0.010		0.008
Primary care	576 (82.1)	126 (17.9)	1 (Reference)		1 (Reference)		1 (Reference)	
Emergency dept	2741 (83.9)	527 (16.1)	0.89 (0.71–1.09)		0.74 (0.59–0.93)		0.73 (0.58–0.92)	
**Food Insecurity (HFSSM)**				<0.001		<0.001		
Food secure	2615 (84.8)	469 (15.2)	1 (Reference)		1 (Reference)		---	---
Food insecure	700 (79.2)	184 (20.8)	1.47 (1.21–1.77)		1.46 (1.19–1.78)		---	---
**Hunger Vital Sign**				<0.001				<0.001
Food secure	2278 (85.3)	393 (14.7)	1 (Reference)		---	---	1 (Reference)	
Food insecure	1037 (80.0)	260 (20.0)	1.45 (1.22–1.73)		---	---	1.44 (1.20–1.73)	
**Interaction (HFSSM × site)**		---	---	---	0.76 (0.45–1.28)	0.297	---	---
**Interaction (Hunger Vital Sign × site)**		---	---	---	---	---	0.87 (0.55–1.40)	0.574

Note: HFSSM = Household Food Security Survey Module, HVS = Hunger Vital Sign. Adjusted ORs were estimated based on logistic regression models, adjusting for maternal age, race, education, marital status, and employment, and child age and birthweight. Site is included in the analyses for food insecurity and food insecurity is included in the analyses for site.

## References

[1] Coleman-Jensen A., Rabbitt M.P., Gregory C., Singh A. (2018). United States Department of Agriculture. Household Food Security in the United States in 2017. https://www.ers.usda.gov/webdocs/publications/90023/err-256.pdf.

[2] Gundersen C., Ziliak J.P. (2015). Food insecurity and health outcomes. Health Aff..

[3] Hanson K.L., Connor L.M. (2014). Food insecurity and dietary quality in US adults and children: A systematic review. Am. J. Clin. Nutr..

[4] Cook J., Frank D., Berkowitz C., Black M., Casey P., Cutts D., Meyers A.F., Zaldivar Z., Skalicky A., Levenson S. (2004). Food insecurity is associated with adverse health outcomes among human infants and toddlers. J. Nutr..

[5] Shankar P., Chung R., Frank D.A. (2017). Association of food insecurity with children’s behavioral, emotional, and academic outcomes: a systematic review. J. Dev. Behav. Pediatr..

[6] Rose-Jacobs R., Black M.M., Casey P.H., Cook J.T., Cutts D.B., Chilton M., Heeren T., Levenson S.M., Meyers A.F., Frank D.A. (2008). Household food insecurity: Associations with at-risk infant and toddler development. Pediatrics.

[7] Jyoti D.F., Frongillo E.A., Jones S.J. (2005). Food insecurity affects school childrens’ academic performance, weight gain, and social skills. J. Nutr..

[8] Whitaker R.C., Phillips S.M., Orzol S.M. (2006). Food insecurity and the risks of depression and anxiety in mothers and behavior problems in their preschool-aged children. Pediatrics.

[9] Portrait F., Teeuwiszen E., Deeg D. (2011). Early life undernutrition and chronic diseases at older ages: The effects of the Dutch famine on cardiovascular diseases and diabetes. Soc. Sci. Med..

[10] Bronte-Tinkew J., Zaslow M., Capps R., Horowitz A., McNamara M. (2007). Food insecurity works through depression, parenting, and infant feeding to influence overweight and health in toddlers. J. Nutr..

[11] Tarasuk V., Cheng J., Oliveira C., Dachner N., Gundersen C., Kurdyak P. (2015). Health care costs associated with household food insecurity in Ontario. Can. Med. Assoc. J..

[12] Eisenmann J.C., Gundersen C., Lohman B.J., Garasky S., Stewart S.D. (2011). Is food insecurity related to overweight and obesity in children and adolescents? A summary of studies, 1995–2009. Obes. Rev..

[13] Drennen C.R., Coleman S.M., Ettinger de Cuba S., Frank D.A., Chilton M., Cook J.T., Cutts D.B., Heeren T., Casey P.H., Black M.M. (2019). Food insecurity, health and development among children under age four years. Pediatrics.

[14] Hager E.R., Quigg A.M., Black M.M., Coleman S.M., Heeren T., Rose-Jacobs R., Cook J.T., Ettinger de Cuba S.A., Casey P.H., Chilton M. (2010). Development and validity of a 2-item screen to identify families at risk for food insecurity. Pediatrics.

[15] Baer T.E., Scherer E.A., Fleegler E.W., Hassan A. (2015). Food insecurity and the burden of health-related social problems in an urban youth population. J. Adolesc. Health.

[16] Gundersen C., Engelhard E.E., Crumbaugh A.S., Seligman H.K. (2017). Brief assessment of food insecurity accurately identifies high-risk US adults. Public Health Nutr..

[17] Gitterman B.A., Chilton L.A., Cotton W.H., Duffee J.H., Flanagan P., Keane V.A., Krugman S.D., Kuo A.A., Linton J.M., McKelvey C.D. (2015). Promoting food security for all children. Pediatrics.

[18] Rasooly I.R., Mullins P.M., Alpern E.R., Pines J.M. (2014). US emergency department use by children, 2001–2010. Pediatr. Emerg. Care.

[19] Children’s HealthWatch The Hunger Vital Sign^™^. http://childrenshealthwatch.org/public-policy/hunger-vital-sign/.

[20] Monette S., Séguin L., Gauvin L., Nikiéma B. (2007). Validation of a measure of maternal perception of the child’s health status. Child Care Health Dev..

[21] Centers for Disease Control and Prevention (1994). National Health and Nutrition Examination Survey 1988–94.

[22] Glascoe F. (1998). Scoring, Administration and Interpretation Guidelines. Collaborating with Parents: Using Parents’ Evaluation of Developmental Status to Detect and Address Developmental and Behavioral Problems.

[23] Glascoe F.P. (2003). Parents’ evaluation of developmental status: How well do parents’ concerns identify children with behavioral and emotional problems?. Clin. Pediatr..

[24] Frank D.A., Casey P.H., Black M.M., Rose-Jacobs R., Chilton M., Cutts D., March E., Heeren T., Coleman S., Ettinger de Cuba S. (2010). Cumulative hardship and wellness of low-income, young children: Multisite surveillance study. Pediatrics.

[25] Black M.M. (2003). Micronutrient deficiencies and cognitive functioning. J. Nutr..

[26] Hurley K.M., Black M.M., Papas M.A., Caulfield L.E. (2008). Maternal symptoms of stress, depression, and anxiety are related to nonresponsive feeding styles in a statewide sample of WIC participants. J. Nutr..

[27] Rose-Jacobs R., Goodhart Fiore J., Ettinger de Cuba S., Black M.M., Cutts D.B., Coleman S.M., Heeren T., Chilton M., Casey P., Cook J. (2016). Children with special health care needs, supplemental security income, and food insecurity. J. Dev. Behav. Pediatr..

[28] Cook J.T., Black M., Chilton M., Cutts D., de Cuba S.E., Heeren T.C., Rose-Jacobs R., Sandel M., Casey P.H., Coleman S. (2013). Are food insecurity’s health impacts underestimated in the US population? Marginal food security also predicts adverse health outcomes in young US children and mothers. Adv. Nutr. Int. Rev. J..

[29] Hunger and Health Feeding America 2017. https://hungerandhealth.feedingamerica.org/explore-our-work/community-health-care-partnerships/addressing-food-insecurity-in-health-care-settings/.

[30] Burkhardt M.C., Beck A.F., Conway P.H., Kahn R.S., Klein M.D. (2012). Enhancing accurate identification of food insecurity using quality-improvement techniques. Pediatrics.

[31] Smith S., Malinak D., Chang J., Perez M., Perez S., Settlecowski E., Rodriggs T., Hsu M., Abrew A., Aedo S. (2017). Implementation of a food insecurity screening and referral program in student-run free clinics in San Diego. Calif. Prev. Med. Rep..

[32] Hassan A., Scherer E.A., Pikcilingis A., Krull E., McNickles L., Marmon G., Woods E.R., Fleegler E.W. (2015). Improving social determinants of health: Effectiveness of a web-based intervention. Am. J. Prev. Med..

[33] Hager K., Cutts D. (2016). Electronic Medical Record (EMR) Based Referrals for Food Insecure Patients to Community Assistance. American Public Health Association Annual Meeting. https://apha.confex.com/apha/144am/meetingapp.cgi/Paper/357527.

[34] Newton A.S., Hartling L., Soleimani A., Kirkland S., Dyson M.P., Cappelli M. (2017). A systematic review of management strategies for children’s mental health care in the emergency department: Update on evidence and recommendations for clinical practice and research. Emerg. Med. J..

[35] Hack C.M., Scarfi C.A., Sivitz A.B., Rosen M.D. (2013). Implementing routine HIV screening in an urban pediatric emergency department. Pediatr. Emerg. Care.

